# Account of the Nardus Indica, or Spikenard

**Published:** 1791

**Authors:** Gilbert Blane


### XVI. *Account of the Nardus Indica, or Spikenard. By* Gilbert Blane, *M. D. F. R. S.* — Vide *Philosophical Transactions of the Royal Society of London,* Vol. LXXX. *for the Year* 1790, Part II.

IN some very judicious reflections, with which this account of the Nardus Indica is prefaced, Dr. Blane expresses his regret that the records of antiquity afford such imperfect descriptions of natural objects, particularly of those of the vegetable kingdom. Most of the writings of the ancients, he observes, have come down to us either mutilated by the accidents

' It is called in Persian, *Tabasheer*; in Hindoo, *Buns-lochun*

' or salt of the Bamboo. It has a peculiar quality of strongly

' adhering to the tongue, and is held in great esteem by the

' natives in a variety of diseases; but I have not yet been able

' to ascertain its virtues from my own experience.

' In a Persian work (the Tofut ul-Monein of Mahomed

' Monein Hoseiny) I have found the following observations

' on this substance, and its supposed medicinal properties, viz.

" It (i. e. the Tabasheer or Buns-lochun) is procured

" from the cavity of the Indian reed or Bamboo; and it is said

" that when, from the violence of the winds, fire takes hold of

" those reed thickets, the Tabasheer is formed of the joints of

" the reeds, which are separated from the ashes thereof. The

" best kind is of a white colour, and of a roundish shape,

" having to the palate a small degree of a rough and biting

" taste. - - - - There is a factitious kind made of burnt bones; but dents of time, or corrupted by unfaithful and ignorant tranfcribers; and he feems to think that the learned works upon profeffional fubjects have been more unfortunate in thefe refpects than works of imagination and general fcience. But even fuppofing the works of Theophraftus, Diofcorides, and the other ancient phyficians and naturalifts, to be extant in their utmoft completenefs and purity, ftill their method of defcribing plants and other natural bodies was fo defective, that very few of them, he obferves, could now be recognized; for we have not only to contend with the obfcurity belong-

" but this has but a fmall degree of bitternefs to the tafte, and " poffeffes no ftrength. - - - The Tabafheer will not diffolve " in water. - - - - It puts a ftop to bilious vomitings and to " the bloody flux. It is alfo of fervice in cafes of palpitation " of the heart, in faintings, and for ftrengthening thofe mem- " bers of the body that are weakened by heat. It is ufeful alfo " for the piles, and for acute or burning fevers, and for puftules " in the mouth (thrufh); and, given with oxymel is of fervice " againft reftleffnefs, melancholy, and hypochondriacal affec- " tions. - - - - The habitual internal ufe of it is prejudicial to " the virile powers. It is alfo faid to be prejudicial to the lungs. " Its correctives are the gum of the pine and honey. The dofe " of it is to the weight of two d'herems or feven mafhás'."

' With the fpecimens of this drug, I fhall alfo fend you a ' piece of the Bamboo unopened, with fome of the falt, or fu- ' gar, in it; from which you will be convinced that the Ta- ' bafheer is not formed by the burning of the bamboo, as the ' author juft now quoted, and others, have fuppofed.'

ing ing to a dead language, in ſo far as the name merely is concerned, but it would be impoſſible, as he juſtly remarks, even in a living language, to perpetuate the knowledge of any object in nature, ſuch as a plant, without ſome deſcription to diſcriminate it from all others. For want of ſuch deſcription the knowledge contained in the writings of the ancient naturaliſts could, he thinks, be of uſe only to their contemporaries and countrymen, who were already acquainted with the objects of it, but could afford no certain information to the ignorant in diſtant countries and future ages. Of all the ancient medicines, he obſerves, there is perhaps none but opium of which the identity can be unqueſtionably aſcertained; of moſt of them little more being ſaid than merely giving their names: ſo that the fruits of the ingenuity and labour of one age have, in a great meaſure, in conſequence of this ambiguity, been loſt to another. Poſterity will, therefore, he adds, be greatly indebted to thoſe induſtrious naturaliſts of the preſent times, who are carrying the deſcription of nature to an unexampled height of improvement.

Dr. Blane has been led, he tells us, to his reflections on this ſubject by an account ſent to him, him, ſome time ago, by his brother in India, of the Spikenard, or *Nardus Indica*, a name familiar in the works of the ancient phyſicians, naturaliſts, and poets; but the identity of which has not hitherto been ſatisfactorily aſcertained. He ſays, in a letter dated Lucknow, December, 1786, that "travelling with the Nabob "Viſier, upon one of his hunting excurſions, "towards the northern mountains, I was ſur-"priſed one day, after croſſing the river Rapty, "about twenty miles from the foot of the hills, "to perceive the air perfumed with an aroma-"tic ſmell; and, upon aſking the cauſe, I was "told it proceeded from the roots of the graſs "that were bruiſed or trodden out of the ground "by the feet of the elephants and horſes of the "Nabob's retinue. The country was wild and "uncultivated, and this was the common graſs "which covered the ſurface of it, growing in "large tufts cloſe to each other, very rank, "and in general from three to four feet in "length. As it was the winter ſeaſon, there "was none of it in flower. Indeed the greateſt "part of it had been burnt down on the road "we went, in order that it might be no impe-"diment to the Nabob's encampments.

"I col-

"I collected a quantity of the roots to be

" dried for ufe, and carefully dug up fome of

" it, which I fent to be planted in my garden

" at Lucknow. It there throve exceedingly,

" and in the rainy feafon it fhot up fpikes about

" fix feet high. Accompanying this I fend

" you a drawing of the plant in flower, and of

" the dried roots, in which the natural appea-

" rance is tolerably preferved.

" It is called by the natives *Terankus*, which

" means literally, in the Hindoo language,

" fever-reftrainer, from the virtues they attri-

" bute to it in that difeafe. They infufe about

" a drachm of it in half a pint of hot water,

" with a fmall quantity of black pepper.

" This infufion ferves for one dofe, and is re-

" peated three times a day. It is efteemed a

" powerful medicine in all kinds of fevers,

" whether continued or intermittent. I have

" not made any trial of it myfelf; but fhall

" certainly take the firft opportunity of doing

" fo.

" The whole plant has a ftrong aromatic
" odour; but both the fmell and the virtues
" refide principally in the hufky roots, which
" in chewing have a bitter, warm, pungent
" tafte, accompanied with fome degree of that
" kind

"kind of glow in the mouth which carda-

"moms occaſion."

Beſides the drawing, a dried ſpecimen, we are told, has been ſent, which was in ſuch good preſervation as to enable Sir Joſeph Banks, P. R. S. to aſcertain it by the botanical characters to be a ſpecies of *Andropogon*, different from any plant that has uſually been imported under the name of Nardus, and different from any of that genus hitherto deſcribed in botanical ſyſtems.

Our author is of opinion, however, that there is great reaſon to ſuppoſe it to be the true *Nardus Indica* of the ancients; for firſt the circumſtance, in the account above recited, of its being diſcovered in an unfrequented country from the odour it exhaled by being trod upon by the elephants and horſes, correſponds, he obſerves, in a ſtriking manner, with an occurrence related by Arrian, in his Hiſtory of the Expedition of Alexander the Great into India. It is there mentioned, lib. vi. cap. 22. that during his march through the deſerts of Gadroſia the air was perfumed by the Spikenard, which was trampled under foot by the army. Secondly, though the accounts of the ancients concerning this plant are obſcure and defective, defective, it is evident, he thinks, that it was a plant of the order of *gramina*, as the term *arista*, so often applied to it, was appropriated by them to the fructification of grains and grasses. The term *spica*, he farther observes, is applied to plants of the natural order *verticillatæ*, in which there are many species of fragrant plants, and the lavender, which being an indigenous one, affording a grateful perfume, was called *Nardus Italica* by the Romans; but we never find the term *arista* applied to these. The poets, as well as the naturalists, constantly apply this term to the true Nardus. In proof of this our author refers to Statius, who calls the Spikenard *odoratæ aristæ*; to Ovid, who, in mentioning it as one of the materials of the phœnix's nest, calls it *Nardi levis arista*; and to a poem on the same subject, ascribed to Lactantius, where the epithet *pubentis* in the expression of *his addit teneras Nardi pubentis aristas*, seems even to point out that it belonged to the genus *andropogon*, a name given to it by Linnæus from this circumstance. Galen, it seems, says, that though there are various sorts of Nardus, the term Ναρδο-σαχυς, or Spikenard, should be applied only to the *Nardus Indica*. It would appear, our author observes, that that the *Nardus Celtica* was a plant of a diffe-rent habit, and is ſuppoſed to be a ſpecies of valeriana. He is aware that Pliny's deſcription of the Nardus Indica does not correſpond with the ſpecimen which is the ſubject of the preſent account, for he ſays it is *frutex radice pingui et craſſa*, whereas this has ſmall fibrous roots. But as Italy is very remote from the native country of this plant, Dr. Blane thinks it rea-ſonable to ſuppoſe, that others, more eaſily pro-curable, uſed to be ſubſtituted for it; and the more ſo, as, according to the author laſt men-tioned, there were nine different plants by which it could be imitated and adulterated. Our au-thor finds a *Nardus Aſſyria* mentioned by Ho-race, and a *Nardus Syriaca* by Dioſcorides, as a ſpecies different from the *Indica*; and both Di-oſcorides and Galen, he adds, by way of fixing more preciſely the country from whence it comes, call it alſo *Nardus Gangites*. Thirdly, Garcias ab Horto, the only writer perhaps, Dr. Blane remarks, who appears to have ſpoken of it in its recent ſtate, from his own obſerva-tions, has given a figure of the roots, or rather the lower part of the ſtalks, which correſponds with our author's ſpecimen. Fourthly, the ſenſible qualities of this ſpecimen are ſuperior, we we are told, to what commonly paſſes for it in the ſhops, being poſſeſſed both of more fragrancy and pungency, which ſeems to account for the preference given to it by the ancients.

It has been a ſubject of inquiry with Mathiolus, whether the roots or ſtalks of this plant were the parts moſt eſteemed by the ancients. The roots of Dr. Blane's ſpecimen, it ſeems, are very ſmall, and poſſeſs ſenſible qualities inferior to the reſt of the plant; yet it is mentioned, in the account above recited, that the virtues reſide principally in the *huſky roots*. It is evident, he obſerves, that by the huſky roots muſt be meant the lower parts of the ſtalks and leaves where they unite to the roots; and he thinks it probable that to a ſlight inaccuracy of this kind is owing the ambiguity on this point that occurs in the ancient accounts.

With regard to the virtues of this plant, Dr. Blane obſerves, that it was highly valued anciently as an article of luxury as well as a medicine. He finds the *unguentum nardinum* ſpoken of as a favourite perfume at the ancient baths and feaſts; and he learns from a paſſage in Horace, that it was ſo valuable, that as much of it as could be contained in a ſmall box of precious ſtone was conſidered as an equivalent for a large veſſel of wine, and a handſome quota for a gueſt to contribute at an entertainment, according to the cuſtom of antiquity :

—— *Nardo vinum merebere*

*Nardi parvus onyx eliciet cadum.*

Its ſenſible qualities, we are told, do not depend on a principle ſo volatile as eſſential oil ; and this, our author thinks, would be a great recommendation of it to the ancients, as its virtues would thereby be more durable, and they were not acquainted with the method of collecting eſſential oils, being ignorant of the art of diſtillation. He finds the fragrance and aromatic warmth of the Nardus to depend on a fixed principle, like that of cardamoms, ginger, and ſome other ſpices. He has tried to extract its virtues by boiling water, by maceration in wine and in proof ſpirits ; but to all theſe menſtrua it yielded them but ſparingly and with difficulty.

As a remedy, both external and internal, it had a high character among the ancients. Dr. Blane finds it one in the liſt of ingredients in all the antidotes, from thoſe of Hippocrates, as given on the authority of Myrepſus and Nicolaus Alexandrinus, to the officinals which have kept their ground till modern times under the names of Mithridate and Venice Treacle. He obſerves that it is recommended by Galen and Alexander Trallian in the dropſy and gravel; that Celſus and Galen employed it both externally and internally in pains of the ſtomach and bowels; and that the latter of theſe phyſicians having been called to attend Marcus Aurelius, when that Emperor was ſeverely afflicted with an acute complaint in the bowels, the firſt remedy he applied was warm oleum nardinum on wool to the ſtomach.

It appears that the natives of India conſider it as an efficacious remedy in fevers; and Dr. Blane obſerves, that its ſenſible qualities promiſe virtues ſimilar to thoſe of other ſimples now in uſe among us in ſuch caſes. Beſides a ſtrong aromatic flavour, it poſſeſſes, we are told, a pungency to the taſte little inferior to the ſerpentaria, and much more conſiderable than the contrayerva. Our author finds it mentioned in a work attributed to Galen, that a medicine, compoſed of this and ſome other aromatics, was found uſeful in long-protracted fevers, which are the caſes, he obſerves, in which medicines of this claſs are employed in modern practice.

The paper is accompanied with an engraved figure of the plant, for which we muft refer our readers to the work itfelf.

